# Knowledge of diabetes among nurses of primary healthcare facilities in China: a cross-sectional survey study

**DOI:** 10.3389/fpubh.2025.1717127

**Published:** 2026-01-07

**Authors:** Dongxue Fei, Jianfeng Wang, Lei Wu, Linhua Pi

**Affiliations:** 1 Department of Metabolism and Endocrinology, The Second Xiangya Hospital, Central South University, Changsha, China; 2 National Clinical Research Center for Endocrine and Metabolic Diseases, Changsha, Hunan, China; 3 Clinical Nursing Teaching and Research Section, The Second Xiangya Hospital of Central South University, Changsha, China; 4 Department of Endocrinology and Metabolism, The Second Affiliated Hospital of Guilin Medical University, Guilin, China; 5 Health Management Center, The Second Affiliated Hospital of Guilin Medical University, Guilin, China; 6 Guangxi Clinical Research Center for Diabetes and Metabolic Diseases, The Second Affiliated Hospital of Guilin Medical University, Guilin, China; 7 Guangxi Health Commission Key Laboratory of Glucose and Lipid Metabolism Disorders, The Second Affiliated Hospital of Guilin Medical University, Guilin, China

**Keywords:** China, cross-sectional studies, diabetes mellitus, knowledge, nursing, primary health care

## Abstract

**Background:**

Nurses with adequate knowledge and experience can reduce hospitalization duration and complications and increase the satisfaction of patients with diabetes. To the best of our knowledge, no study has assessed the current state of diabetes knowledge among nurses in PHC facilities in China.

**Methods:**

This cross-sectional study was conducted using self-administered questionnaires among nurses affiliated with 12 township health centers in Pingjiang City, Hunan Province from October 4 to December 15, 2024. Knowledge levels were determined by calculating questionnaire scores and analyzed using the independent-samples *t*-test and one-way analysis of variance (ANOVA) test. And factors affecting knowledge levels were analyzed using multiple linear regression models. Data were analyzed using SPSS version 25.

**Results:**

A total of 223 nurses completed the survey. The mean diabetes knowledge score was 16.94 ± 3.27 (out of 23 points). Regression analyses indicated that sex, professional title, and department significantly influenced knowledge scores (*p* < 0.05).

**Conclusion:**

Nurses from PHC facilities in China demonstrated a moderate level of diabetes-related knowledge. Inappropriate knowledge negatively affects the care of diabetic patients. Health authorities and hospital administrators should implement targeted interventions to improve nurse education in PHC facilities to facilitate the provision of high-quality and comprehensive diabetes care.

## Introduction

Diabetes mellitus (DM) is a chronic metabolic disease characterized by hyperglycemia and associated complications, which poses a global public health concern. According to the latest estimates of the International Diabetes Federation (IDF), 589 million (10.5%) adults worldwide are expected to have DM in 2024, which is further projected to increase to 853 million by 2050. Approximately 3.4 million adult deaths, which accounts for 9.3% of all deaths globally, are attributed to DM and its complications (1). DM is also associated with enormous healthcare costs, which place a heavy burden on patients, their families, and the society (2).

Due to population aging and lifestyle changes, China has become the country most affected by DM, with 148 million individuals living with diabetes (1). The burden of NCDs that China now faces is monumental, especially diabetes (3) and hypertension (4). Acknowledging the increasing pressure exerted by NCDs, the Healthy China 2030 initiative of the Chinese government emphasizes the role of the primary healthcare (PHC) system as the frontline in addressing these chronic diseases (5). In China, most patients with type 2 DM (T2DM) receive healthcare services in PHC facilities (6), which are organized into clinical- and center-level institutions, including community health service stations and centers in urban areas, village clinics, and township health centers in rural areas.

To improve the quality of PHC basic services, the Chinese government promoted a basic public health package in 2009, which expanded to 14 categories in 2017, including five related to diabetes management and control (7). Despite these efforts, there are substantial gaps in optimal care for people with T2DM in China. A recent study revealed that the achievement of guideline-recommended hemoglobin A1c, blood pressure, and low-density lipoprotein cholesterol, which are collectively referred to as the ABC targets, was only 5.6% (8).

The IDF has called for better-trained nurses to address the challenges of diabetes care (1). Nurses play a vital role in managing patients with DM. Previous studies have shown that nurses with adequate knowledge and practice can effectively shorten the length of hospitalization, reduce complications, and increase patient satisfaction (9, 10). However, previous studies have indicated varying levels of knowledge regarding DM management among nurses in different countries. For instance, gaps have been identified in Australia (11), Saudi Arabia (12), Ethiopia, particularly on diabetic foot care (13), and Iran, particularly on nutritional management of diabetes mellitus (14). Studies conducted in China reported a limited understanding of diabetes among non-endocrinology nurses in tertiary general (15) and primary care hospitals (16).

To the best of our knowledge, no study has assessed the current state of diabetes knowledge among nurses in PHC facilities in China. This study used a questionnaire to evaluate the diabetes knowledge of nurses from PHC facilities and analyze relevant influencing factors. Understanding these gaps and issues would aid in making policies aimed at improving diabetes care in primary care settings.

## Methods

### Study design and participants

This descriptive cross-sectional study used a convenience sampling approach and was conducted among nurses affiliated with 12 township health centers in Pingjiang City, Hunan Province, from October 4 to December 15, 2024. Briefly, lists of all primary care-based nurses were attained from all 12 township health centers, the umbrella organization of all general practices, in Pingjiang City. Finally, 768 PHC nurses were identified in Pingjiang City. All nurses directly engaged in the care and management of patients with DM who provided informed consent were included in the study. The exclusion criteria were as follows: (1) holding only administrative roles; (2) having a nurse practitioner license with <1 year of practical experience; and (3) working in support departments, such as pharmacy and laboratory services.

Using OpenEpi web-based calculator, with a significance level of 95, 5% absolute precision, and a population size of 800, the minimum sample size computed was 195 participants. Considering a 10% contingency, the minimum sample size calculated was 216 participants. A simple random sampling method was used to select 400 nurses from 12 health centers. Ultimately, 223 participants completed the questionnaire, resulting in a response rate of 55.75%.

### Tool of the study

The self-administered questionnaire contained two sections. The first section gathered demographic and practice-related data, including sex, age, education level, professional title, years of experience, and department. The second section was adapted from the Michigan Diabetes Knowledge Test (DKT), which was developed by a panel of experts from the University of Michigan Diabetes Research and Training Center in 1990 (17). This test has been reported to have good reliability and validity (18) and has been widely used in previous studies to assess DM knowledge (18–20). Permission to use and modify the instrument accordingly was obtained from the primary authors. Two experts in endocrinology evaluated the questionnaire items for difficulty and clarity. A pretest was conducted among 20 nursers to test the reliability and improve the clarity and interpretability of the questionnaire. Reliability of the instrument was assessed through internal consistency method after it was completed by 20 nursers. The reliability of the instrument was approved and the overall Cronbach’s alpha was 0.865. The DKT contains two subscales: a 14-item general diabetes subscale and a 9-item insulin-use subscale. Each item has three or four options, with only one correct answer. Questions were scored using a two-point scale (1 for correct and 0 for incorrect), resulting in total scores ranging from 0 to 23, with higher scores indicating greater knowledge of diabetes.

### Data collection

The questionnaire was distributed through an online survey platform, Questionnaire Star, which provides functions equivalent to Amazon Mechanical Turk, including surveys, examinations, assessments, and voting. The final questionnaire was formatted electronically with standardized instructions. We sent the survey link to eligible participants via text message or WeChat. Participants could access the survey by clicking the link, and responses were automatically recorded upon completion. All responses were anonymous. To avoid duplicate submissions, Questionnaire Star ensured that the same IP address, mobile phone, tablet, or computer could only submit one entry.

### Ethical approval

The study adhered to the Declaration of Helsinki, and ethical approval was obtained from The Second Affiliated Hospital of Guilin Medical University. At the beginning of the online questionnaire, the voluntary nature of the study and confidentiality of responses were described, and informed consent was obtained from all participants.

### Statistical analysis

Data were extracted and analyzed using SPSS version 25.0 (IBM Corporation, Armonk, NY, USA). Descriptive data were presented as numbers, percentages, means, and standard deviations, depending on variable type. Associations between the characteristics and knowledge level of nurses were evaluated using ANOVA test and t-test, as appropriate. Multiple linear regression models were used to examine the association between predictor variables and KAP scores. All significant factors in the univariate analyses were included in the multiple linear regression analysis. Statistical significance was set at *p* < 0.05.

## Results

### Participant characteristics

This study was conducted between October 4 and December 15, 2024. Of the 400 nurses approached, 223 agreed to participate in our study (response rate: 55.8%). Table 1 presents the demographics and characteristics of the participants. Most participants were female (*n* = 197, 88.4%). The mean (standard deviation) age of the nurses was 29.5 ± 6.5 years, with most nurses ≤30 years (*n* = 153, 68.6%). Regarding education, 56.6% held an associate or below degree and 160 (71.7%) were junior nurses. Most nurses had 5–10 years of work experience (39.5%) and were employed in internal medicine departments (75.7%).

**Table 1:** Demographic and characteristics of participants (*N* = 223).

Characteristics	Mean±SD	Number (n)	Percentage (%)
Sex			
Male		26	11.6%
Female		197	88.4%
Age (years)	29.5 ± 6.5		
≤30		153	68.6%
>30		70	31.4%
Education level			
Associate degree and below		126	56.5%
Bachelor degree		97	43.5%
Professional titles			
Junior nurse		160	71.7%
Change nurse		63	28.3%
Duration of practice (years)			
<5		70	31.4%
5–10		88	39.5%
≥10		65	29.1%
Department			
Internal medicine		169	75.7%
Surgery		54	24.3%

SD, standard deviation.

### Diabetes knowledge level of nurses in PHC facilities

The average correct response rate among the nurses was 73.7%. Table 2 presents the distribution of diabetes knowledge scores. Regarding general knowledge, nurses demonstrated a good knowledge on the optimal method for blood glucose testing (77.4%), treatment of hypoglycemia (75.3%), effects of exercise and infection on blood glucose (90.1 and 88.3%, respectively), best practices for foot care (87.0%), causes of numbness and tingling (93.3%), and complications of DM (87.9%). However, knowledge on other general topics was found to be modest or poor.

**Table 2:** Diabetes knowledge level of nurses in PHC facilities (*N* = 223).

Number	Diabetes knowledge test items	Correct *n* (%)	Incorrect n (%)
	General knowledge		
1	The definition of diabetes diet	131 (58.7%)	92 (41.3%)
2	Foods highest in carbohydrate content	118 (52.9%)	105 (47.1%)
3	Foods highest in fat content	102 (45.7%)	121 (54.3%)
4	The definition of sugar-free foods	105 (47.1%)	118 (52.9%)
5	Glycosylated hemoglobin (hemoglobin A1) test definition	122 (54.7%)	101 (45.3%)
6	The optimal method for blood glucose testing	166 (74.4%)	57 (25.6%)
7	Influence of sugar-free fruit juice on blood glucose	144 (64.6%)	79 (35.4%)
8	Treatment of hypoglycemia	168 (75.3%)	55 (24.7%)
9	Influence of exercise on blood glucose	201 (90.1%)	22 (9.9%)
10	Influence of infection on blood glucose	197 (88.3%)	26 (11.7%)
11	The best behavior of foot care of diabetic patients	194 (87.0%)	29 (13.0%)
12	The consequences of high-fat foods among diabetic patients	99 (44.4%)	124 (55.6%)
13	Causes of numbness and tingles in among diabetic patients	208 (93.3%)	15 (6.7%)
14	Complications of DM	196 (87.9%)	27 (12.1%)
	Insulin Knowledge		
15	Symptoms of ketoacidosis	139 (62.3%)	84 (37.7%)
16	Optimal management of flu among diabetic patients	175 (78.5%)	48 (21.5%)
17	Action time of intermediate-acting insulin	162 (72.6%)	61 (27.4%)
18	Proper action if diabetic patient forgot to take his/her insulin prior breakfast	201 (90.1%)	22 (9.9%)
19	Proper action if diabetic patient having symptoms of hypoglycemia	192 (86.1%)	31 (13.9%)
20	Causes of hypoglycemia	203 (91.0%)	20 (9.0%)
21	Influence of insulin injection without eating breakfast	214 (96.0)	9 (4.0%)
22	Causes of hyperglycemia	164 (73.5%)	59 (26.5%)
23	Causes of insulin reaction	177 (79.4%)	46 (20.6%)
	Overall	73.7%	26.3%

PHC, primary healthcare; DM, diabetes mellitus.

Regarding insulin-related knowledge, nurses also demonstrated good knowledge except for the symptoms of ketoacidosis (62.3%), onset time of intermediate-acting insulin (72.6%), and causes of hyperglycemia (73.5%).

### Factors associated with DM knowledge

Table 3 presents the associations between DM knowledge and participant characteristics. Univariate analysis revealed significant differences in DM knowledge based on sex, professional title, years of experience, and department (*p* < 0.001, *p* = 0.036, and *p* = 0.006, respectively).

**Table 3:** Factors associated with DM knowledge (*N* = 223).

Variable	*N*	Mean±SD	*p*-value
Sex			<0.001^a^
Male	25	14.80 ± 2.72	
Female	198	17.21 ± 3.24	
Age (years)			0.74^a^
≤30	153	16.75 ± 3.38	
>30	70	17.37 ± 2.99	
Education level			0.372^a^
Associate degree and below	126	16.77 ± 3.49	
Bachelor degree	97	17.16 ± 2.97	
Professional titles			<0.001^a^
Nurse	160	16.41 ± 3.40	
Nursing Assistant	63	18.29 ± 2.48	
Duration of practice (years)			0.036^b^
<5	70	16.52 ± 3.74	
5–10	88	16.62 ± 3.24	
≥10	65	17.81 ± 2.57	
Department			0.006^a^
Internal medicine	169	17.28 ± 3.08	
Surgery	54	15.89 ± 3.62	

a, *t*- test; b, ANOVA test. DM, diabetes mellitus; SD, standard deviation.

Furthermore, multiple linear regression analysis showed that sex, professional title, and department influenced DM knowledge scores. Females had a higher level of knowledge than males, and knowledge levels were positively associated with professional titles. Nurses from internal medicine departments possessed higher knowledge levels than those from surgery departments (Table 4).

**Table 4:** Multiple linear regression identifying factors associated with DM knowledge.

Independent variable	*b*	Sb	*b*’	*t*	*p*-value
Constant	14.511	0.681	-	21.299	<0.001
Sex	2.529	0.652	0.245	3.881	<0.001
Professional titles	1.597	0.541	0.220	2.950	0.004
Duration of practice (years)	0.063	0.315	0.015	0.199	0.842
Department	−1.355	0.485	−0.178	−2.794	0.006

*F* = 9.520, *P* < 0.001, *R*^2^ = 0.149, adjusted *R*^2^ = 0.133. DM, diabetes mellitus.

## Discussion

This study aimed to investigate the knowledge of nurses in PHC facilities regarding the care and management of patients with diabetes and to identify influencing factors that could aid in policy making for improving diabetes management in primary care settings. The findings revealed that nurses in PHC facilities had a moderate level of knowledge about diabetes, with significant differences based on sex, professional title, and department.

Due to population aging and lifestyle changes, China has become the country most affected by DM, placing a heavy burden on its healthcare system (1). Optimal diabetes care requires healthcare providers, such as nurses, and patients to have adequate knowledge and skills regarding DM and its management. It is critical that nurses have a sufficient understanding of all aspects of DM management to inform and support their patients in effectively managing their condition. To the best of our knowledge, this is the first study conducted in a primary care setting to evaluate the DM knowledge of nurses. Previous studies have indicated that both primary care physicians (21, 22) and patients with diabetes had relatively low diabetes knowledge. Consistent with previous studies, the results of this study indicated that nurses in PHC facilities demonstrated a moderate level of diabetes knowledge. Furthermore, this finding aligns with studies conducted among non-endocrinology nurses in primary hospitals (16) and tertiary general hospitals in China (15). However, the higher scores observed in our study may be attributed to the focus of the questionnaire, which evaluated basic DM knowledge, excluding content from the latest guidelines and advancements in diabetes-related technologies, thereby reducing the difficulty of the assessment. Our findings are also comparable to those of studies conducted among PHC nurses in New Zealand (23) and Australia (11).

The management of diabetes is complex and multifaceted, encompassing diet and nutrition, exercise, blood glucose monitoring, and complication screening (24). Regarding general DM knowledge, the nurses in this study showed poor knowledge on diabetes-related diets and nutrition, consistent with studies conducted in Australia (11), Sub-Saharan Africa (25), Morocco (26) and Poland (27) and the United States (28). These studies demonstrated that nurses have inadequate knowledge on various aspects of nutrition management for diabetes, such as providing dietary advice, meal planning, and questions related to the sources of carbohydrates. Nutrition is one of the most common non-pharmaceutical therapeutic strategies for diabetes care, and the poor performance of nurses may be due to the fact that dietary prescriptions for patients with DM are usually prescribed by dietitians in comprehensive hospitals. In addition, dietitian and nutrition-related training are lacking in PHC settings.

Contrary to studies from the United States (28), United Kingdom (29), and Jordan (30), which reported low insulin-related knowledge among nurses, our results showed higher knowledge scores that may be attributed to the time of these study. The results of the current study indicated that nurses in PHC facilities demonstrated good knowledge, except for the symptoms of ketoacidosis (62.3%), duration of intermediate-acting insulin (72.6%), and causes of hyperglycemia (73.5%). These gaps may be attributed to the following reasons: (1) ketoacidosis, a life-threatening condition, is often admitted to a comprehensive hospital for treatment, limiting exposure of PHC nurses (31); (2) insulin may not always be accessible in PHC facilities, reducing the knowledge and competencies of primary care professionals in insulin use (32); and (3) nurses may not be directly responsible for treating diabetes, leading to limited practical knowledge. These findings highlight the need for training programs focusing on nutritional and insulin management knowledge, and national guidelines should be proposed to facilitate the provision of high-quality and comprehensive diabetes care.

This study also found that higher DM knowledge among nurses in PHC facilities was related to female sex, higher professional titles, and working in internal medicine departments. This finding indicated a significant knowledge gap between male and female nurses, similar to a previous study from primary hospitals in China (13), possibly because nursing is a female-dominated profession, and women may have greater involvement in diabetes care, increasing their motivation to acquire knowledge regarding diabetes. Nurses with higher professional titles are typically required to engage in continuing education, which may explain their greater knowledge (13). Nurses from internal medicine departments scored higher than their counterparts from surgery departments, which is supported by a previous study that reported that intensive care unit nurses scored lowest on diabetes knowledge (15). This finding may be related to the nature of their position, wherein internal medicine nurses have more opportunities to care for patients with diabetes. Therefore, health authorities and hospital administrators should prioritize diabetes knowledge training programs for surgical nurses.

## Limitations

This study has some limitations. First, this study was restricted to one geographic region in Central China, and our results may not be generalizable to all nursers across China. Second, this was a cross-sectional study, therefore, only associations could be determined but not causal relationships. Third, the use of a self-report survey may have introduced recall and social desirability biases, with more respondents reporting positive attitudes toward diabetes.

## Conclusion

This study found that nurses from PHC facilities in China had moderate levels of diabetes knowledge. Female nurses with higher professional titles had higher levels of knowledge about diabetes, and nurses from internal medicine departments scored higher than their counterparts from surgery departments. Identifying knowledge gaps in diabetes care and management is the first step in providing tailored measures and improving DM care. Health authorities and hospital administrators should implement targeted interventions to improve the diabetes knowledge level of nurses from PHC facilities to facilitate the provision of high-quality and comprehensive diabetes care.

## Data Availability

The raw data supporting the conclusions of this article will be made available by the authors, without undue reservation.
